# Endosome-mediated endocytic mechanism replenishes the majority of synaptic vesicles at mature CNS synapses in an activity-dependent manner

**DOI:** 10.1038/srep31807

**Published:** 2016-08-18

**Authors:** Joohyun Park, Oh Yeon Cho, Jung Ah Kim, Sunghoe Chang

**Affiliations:** 1 Department of Physiology and Biomedical Sciences, Seoul National University College of Medicine, Seoul 110-799, South Korea; 2 Biomembrane Plasticity Research Center, Seoul National University College of Medicine, Seoul 110-799, South Korea; 3 Interdisciplinary Program in Neuroscience, Seoul National University College of Medicine, Seoul 110-799, South Korea; 4 Neuroscience Research Institute, Medical Research Center, Seoul National University College of Medicine, Seoul 110-799, South Korea.

## Abstract

Whether synaptic vesicles (SVs) are recovered via endosome-mediated pathways is a matter of debate; however, recent evidence suggests that clathrin-independent bulk endocytosis (CIE) via endosomes is functional and preferentially replenishes SV pools during strong stimulation. Here, using brefeldin-A (BFA) to block CIE, we found that CIE retrieved a minority of SVs at developing CNS synapses during strong stimulation, but its contribution increased up to 61% at mature CNS synapses. Contrary to previous views, BFA not only blocked SV formation from the endosome but also blocked the endosome formation at the plasma membrane. Adaptor protein 1 and 3 (AP-1/3) have key roles in SV reformation from endosomes during CIE, and AP-1 also affects bulk endosome formation from the plasma membrane. Finally, temporary blocking of chronic or acute neuronal activity with tetrodotoxin in mature neurons redirected most SV retrieval to endosome-independent pathways. These results show that during high neuronal activity, CIE becomes the major endocytic pathway at mature CNS synapses. Moreover, mature neurons use clathrin-mediated endocytosis and the CIE pathway to different extents depending on their previous activity; this may result in activity-dependent alterations of the SV composition which ultimately influence transmitter release and contribute to synaptic plasticity.

Typical central synapses contain a limited number of synaptic vesicles (SVs), and to continuously release neurotransmitters in response to repetitive stimulation, the SVs need to be quickly replenished through endocytosis and recycling. Among multiple recycling mechanisms proposed, besides the transient opening of fusion pores called *kiss-and-run*, two distinct modes of retrieval after full fusion with the plasma membrane are known: clathrin-mediated endocytosis (CME) and clathrin-independent bulk endocytosis (CIE)^1^,^2^. CME uses adaptor protein 2 (AP-2) to retrieve SVs during mild stimulation, whereas during intense activity, CIE is used^3^,^4^,^5^,^6^. Unlike CME, which deals with single SVs, activity-dependent bulk endocytosis invaginates a large region of the plasma membrane, creating bulk endosomal intermediates from which SVs can be budded via AP-1 and AP-3 dependent mechanisms^7^,^8^,^9^,^10^,^11^.

Despite many years of research, however, a conclusive answer for the role of clathrin and AP-2 in SV recycling has remained elusive. Indeed, a recent study showed that clathrin is not essential for SV retrieval at the membrane, in particular during high frequency stimulation. Instead, dynamin 1/3 and endophilin-dependent clathrin-independent endocytosis forming endosome-like vacuoles are functional, and clathrin and AP-2 mediate SV formation from endocytosed endosomal structures, suggesting stimulation-dependent modulation of endocytic mechanisms at central nervous system (CNS) synapses^12^. In addition, *Watanabe et al.* also have reported that akin to bulk endocytic retrieval, another mode of endocytosis called ultrafast endocytosis retrieves single, large endocytic vesicles directly adjacent to presynaptic densities as fast as 50 ~ 100 ms after SV fusion in response to a single stimulus or during mild stimulation^13^. They further showed that clathrin is not required for ultrafast endocytosis but is required to generate SVs from the endosomes at physiological temperature (34 ~ 37 °C). At room temperature (~22 °C), however, CME functions at the plasma membrane^14^. Therefore, the frequency of stimulation and/or temperature could be the regulating factor that determines whether the CME and CIE pathway is used.

Here, we examined how neurons use CME and CIE during neuronal maturation in primary cultured hippocampal neurons. Our data show that during strong stimulation, early developing neurons retrieve only 1/3 of SVs via CIE; however, the contribution of CIE to SV endocytosis increases as neurons mature and reaches as much as 2/3 of SVs in fully mature neurons. We found that CIE captures SVs through AP-1/3 dependent mechanisms, and that the extent of CIE is not fixed because chronic or acute inactivity induced by tetrodotoxin (TTX) forces most SVs into the endosome-independent recycling pathway.

## Results

To estimate the contribution of the endosome-mediated CIE mode to SV recycling, we used BFA which is known to inhibit protein transport from the endoplasmic reticulum to the Golgi complex indirectly by preventing the formation of coat protein 1 (COP1)-mediated transport vesicles^15^,^16^. Although the target of BFA in endosomal systems is not well-defined, AP-1/-3-dependent SV recycling on endosomal membranes via CIE is known to be sensitive to BFA^7^,^8^.

**Figure 1:**
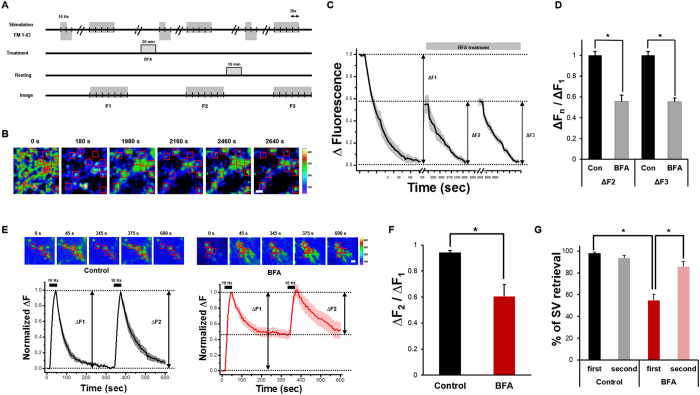
SVs are solely retrieved by BFA-insensitive endocytic pathway in the presence of BFA. (**A**) Hippocampal neurons at DIV19 were loaded with FM 1–43 by field stimulation (300 APs at 10 Hz) and kept for an additional 30 s after the stimulation to label all endocytosed vesicles. After 10 min of resting period, a stimulus of 1,200 APs at 10 Hz was given to unload FM 1–43. After the first round of dye loading/unloading, neurons were treated with 10 μg/ml BFA for 30 min (*n* = 220 neurons from 9 independent coverslips). (**B**) Representative fluorescent time-lapse images of presynaptic boutons treated with FM 1–43. Individual regions were selected by hand and rectangular regions of interest (red) were drawn around the synaptic boutons, then average intensities were calculated. Large puncta, which are clusters of synapses, were excluded from the selection procedure. Right: heat-map for pseudo-colored fluorescence intensity. Scale bar: 1 μm. (**C)** The extent of SV turnover during the first (ΔF1), second (ΔF2) and third cycle (ΔF3). All values were normalized to ΔF1. Error bars: SEM. (**D)** ΔF2 and ΔF3 over ΔF1, respectively. Data are presented as means ± SEM *p < 0.01 (Student’s *t*-test). (**E)** Representative fluorescent time-lapse images of SypHy transfected presynaptic boutons. Average SypHy fluorescence intensity profiles of the control (black) and BFA treated neurons (red) that were stimulated with 300 APs at 10 Hz twice. ΔF1 = (*F*_*max of the first stimulation*_ − *F*_*0 before the first stimulation*_), ΔF2 = (*F*_*max of the second stimulation*_ − *F*_*0 before the second stimulation*_). All values were normalized to ΔF1. Error bars: SEM (*n* = 83 neurons from 9 independent coverslips for control group, *n* = 69 neurons from 6 independent coverslips for BFA treatment group). Scale bar: 1 μm. (**F)** The ratio of ΔF2 over ΔF1. *p < 0.01 (Student’s *t*-test). (**G**) % of SV retrieval was calculated by (*F*_*max of each stimulation*_ − *F*_*300 s after each stimulation*_)/(*F*_*max of each stimulation*_ − *F*_*0 before each stimulation*_) × 100. Data are presented as means ± SEM *p < 0.01 (Student’s *t*-test).

First, we used FM 1–43 to label routes of SV retrieval by endocytosis. FM 1–43 is a green fluorescent styryl membrane dye widely used to study SV recycling kinetics^17^. We found that in BFA-treated neurons but not in DMSO treated neurons, FM 1–43 uptake upon stimulation with 300 action potentials (APs) at 10 Hz was reduced by approximately half (Fig. 1A–D and Supplemental Fig. 1), which was consistent with the results from a previous study^7^. We further found that this endocytic defect persisted during another round of FM 1–43 loading-unloading (Fig. 1C,D), confirming that CIE was induced by our stimulation protocol and was blocked by BFA treatment.

In further experiments, to measure the endocytic behavior of SVs, we used synaptophysin fused with pHluorin (SypHy), a modified green fluorescent protein (GFP) with high pH sensitivity^19^,^20^ (Supplemental Fig. 2A). At rest, the fluorescence of SypHy is quenched by the acidic intraluminal pH (pH ~ 5.5). Upon stimulation, vesicles fuse with the plasma membrane exposing their intraluminal proteins to the basic extracellular medium (pH 7.4), resulting in an increase in fluorescence. During endocytosis, the rapid reacidification of vesicles leads to a decrease in SypHy fluorescence as the pHluorin molecule is quenched once again. A typical response from presynaptic boutons expressing SypHy in dissociated hippocampal neurons to a train of stimuli is shown in Supplemental Fig. 2.

To examine the kinetics of SV retrieval in more detail, we used a double stimulation protocol. We stimulated the neurons with 300 APs at 10 Hz and re-stimulated them with the same stimulus after 5 min. In the control group without BFA, the fluorescence change after the second stimulation (∆F_2_) was comparable to that seen after the first stimulation (∆F_1_), and the exocytosed SVs were retrieved fully after both stimuli (97.3 ± 1.8% for the first stimulation and 92.5 ± 2.3% for the second stimulation; Fig. 1E–G).

In the presence of BFA, however, the fluorescence of SypHy failed to decrease to the initial level after the first stimulation (58.2 ± 5.8% of released, Fig. 1E–G), indicating an incomplete retrieval of the released SVs. Intriguingly, after the second stimulation, the fluorescence of SypHy increased up to the peak value of the first stimulation and then decreased back to its initial value before the second stimulation. This indicated that the SVs released by the second stimulation were almost completely retrieved by endocytosis (85.1 ± 5.6% of released; Fig. 1E–G), suggesting that the SVs retrieved after the second stimulation in the presence of BFA were those that were retrieved by the BFA-insensitive retrieval pathway after the first stimulation.

The incomplete decrease of SypHy fluorescence after the first stimulation in the presence of BFA, however, remained hard to understand because most previous studies have suggested that BFA does not arrest the formation of endosomes during CIE but stops subsequent SV budding (see ref. 8). Again, even if budding was arrested, one would imagine that the endosomes would still be acidified, and thus, the SypHy signal would decrease to the pre-stimulation level. To explain this enigma, we examined the following two possibilities: In scenario-1, BFA does not block the budding of bulk endosomes from the plasma membrane but inhibits their subsequent re-acidification, and in scenario-2, BFA actually blocks the budding of bulk endosomes from the plasma membrane (that is, the inside of the bulk endosomes should be accessible from outside the cell).

**Figure 2:**
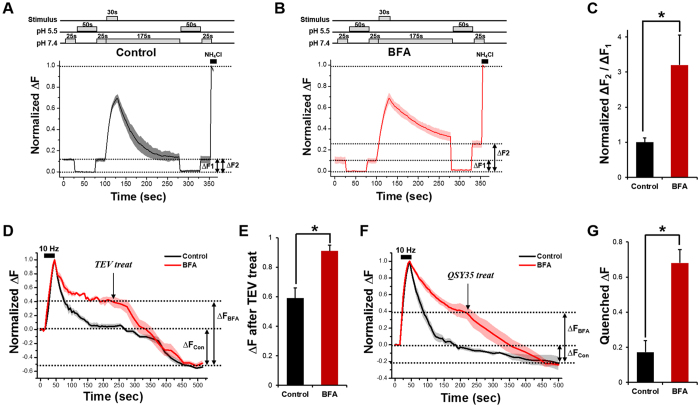
BFA also blocks the budding of the bulk endosome off from the plasma membrane. **(A,B)** Average traces of SypHy fluorescence intensity in the control and BFA-treated neurons during the pH exchange experiments. The extracellular solution of pH 5.5 rapidly quenched all surface-bound SypHy. After 50 s, the pH of the extracellular solution was changed back to 7.4. After the subsequent stimulus for 30 s at 10 Hz, the extracellular solution was changed again to pH 5.5, remained for 50 s to quench all exposed SypHy, and changed back to 7.4. Net SypHy fluorescence changes were obtained by subtracting the average intensity at pH 5.5 (F_5.5_) from the intensity of each frame (F_t_) for individual boutons, and they were normalized to the peak SypHy response in the presence of NH_4_Cl. (**C)** Average degree of quenching post-stimulus (ΔF2) compared to pre-stimulus (ΔF1) was 100 ± 12.1% in control neurons (*n* = 76 neurons from 5 independent coverslips) and 320 ± 84.6% in BFA-treated neurons (*n* = 92 neurons from 5 independent coverslips). Data are presented as means ± SEM *p < 0.05 (Student’s *t*-test). **(D,E)** Average degree of decreased fluorescence after TEV treatment in the presence or absence of 10 μg/ml BFA. Data are presented as means ± SEM *p < 0.05 (Student’s *t*-test). (**F)** QSY35 was treated at 5 min after the end of the stimulation. Fluorescence of the BFA-treated neurons was decreased down to a level comparable to that obtained in control neurons (*n* = 26 neurons from 4 independent coverslips for control, *n* = 36 neurons from 3 independent coverslips for BFA treatment). (**G)** Average degree of quenched fluorescence in the presence or absence of 10 μg/ml BFA after QSY35 treatment. Data are presented as means ± SEM *p < 0.01 (Student’s *t*-test).

To test these possibilities, we first did re-acidification experiments (Fig. 2A). If scenario-1 is true, then ΔF1 (before stimulation) and ΔF2 (after stimulation) should be the same in the BFA-treated neurons (ΔF1 = ΔF2 and Supplemental Fig. 3A,B). If scenario-2 is true, a second application of pH 5.5 after stimulation should decrease SypHy fluorescence all the way to zero (ΔF1 < ΔF2 and Supplemental Fig. 3A,B). The results showed that ΔF2 was ~3-fold higher than ΔF1 in the presence of BFA, which validates scenario-2 (Fig. 2A–C).

To test this conclusion further, we performed tobacco etch virus (TEV) protease cleavage experiments. We used a synaptobrevin-TEV-pHluorin construct which has a TEV protease cleavage site between the synaptobrevin-2 and ecliptic-pHluorin moiety. External TEV protease would eliminate the ecliptic pHluorin, leading to a gradual loss of fluorescence only when this protein is in the plasma membrane or accessible from outside the cell (Supplemental Fig. 3C,D). If scenario-1 is true, TEV protease should not have access to the cytosol of the bulk endosomes; thus, the resulting amount of fluorescence loss in the BFA-treated neurons should be the same as that in the control neurons (ΔF_Con_ = ΔF_BFA_). If scenario-2 is true, TEV protease could cleave the ecliptic pHluorin not only in the plasma membrane but also inside the bulk endosomes, and ΔF_BFA_ should be larger than ΔF_Con_. We found that in the presence of BFA, the loss of fluorescence in TEV treated neurons was much larger than that in the control (Fig. 2D,E). The results again support scenario-2.

Finally, we used a fluorescence-quencher, QSY35, a diarylrhodamine derivative that efficiently quenches GFP fluorescence^21^. Again, if scenario-2 is true, QSY35 should have access to the cytosol of the bulk endosomes and quench the ecliptic pHluorin inside the bulk endosomes as well as in the plasma membrane, and ΔF_BFA_ should be larger than ΔF_Con_. The results show that in the presence of BFA, the QSY35 treatment resulted in larger loss of fluorescence than that of the control, which supports scenario-2 (Fig. 2F,G).

Therefore, all our experiments indicate that in addition to its effect on SV budding from the endosome, BFA also blocks the budding of bulk endosomes from the plasma membrane.

### BFA-sensitive CIE becomes a major endocytic route when neurons mature

**Figure 3:**
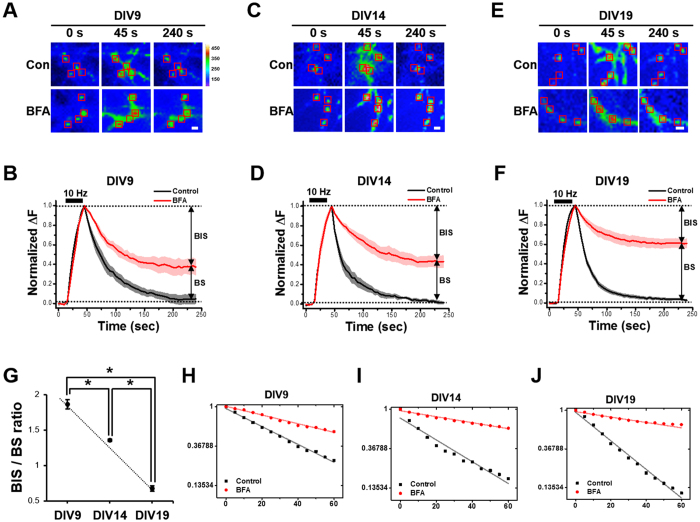
The BFA-sensitive SV retrieval becomes the major pathway as neuronal maturation proceeds. **(A,C,E)** Representative fluorescent time-lapse images of SypHy transfected neurons at DIV9 (**A**), DIV14 (**C**) and DIV19 (**E**) in response to a stimulus of 300 APs at 10 Hz. Rectangular regions of interest were drawn around the synaptic boutons, then average intensities were calculated. Con : without BFA; BFA : 30 min treatment of 10 μg/ml BFA. Heat-map for pseudo-colored fluorescence intensity is shown. Scale bar: 1 μm. (**B,D,F)** Average SypHy fluorescence intensity profiles of the boutons from DIV9 (**B**), DIV14 (**D**) and DIV19 (**F**) neurons before (black) or after (red) the 30 min treatment of 10 μg/ml BFA with a stimulus of 300 APs at 10 Hz. The decay of SypHy was fitted by a double exponential function. Note that regardless of maturation, the SVs retrieval is sensitive to BFA although the degrees of sensitivity are different (*n* = 94 neurons from 4 independent coverslips for DIV9, *n* = 190 neurons from 4 independent coverslips for DIV14, *n* = 455 neurons from 4 independent coverslips for DIV19). (**G)** The ratio of BFA-insensitive SV (BIS) to BFA-sensitive SV (BS) is 1.86 ± 0.06 for DIV9, 1.36 ± 0.01 for DIV14, and 0.67 ± 0.04 for DIV19 neurons. (**H–J)** Semilog plots of the traces for DIV9 **(H)**, DIV14 **(I)** and DIV19 **(J)** neurons are shown (control: *k* = 0.023 s^−1^ for DIV9; *k* = 0.028 s^−^1 for DIV14; *k* = 0.037 s^−1^ for DIV19; BFA-treated: 0.011 s^−1^ for DIV9; 0.008 s^−1^ for DIV14 and 0.006 s^−1^ for DIV19). Data are presented as means ± SEM *p < 0.01 (ANOVA and Tukey’s HSD *post hoc* test).

We next tested how the relative usage of CME and CIE is regulated during neuronal maturation. We transfected neurons at 7 days *in vitro* (DIV) with SypHy and measured the endocytic behavior at DIV9 in the presence and absence of BFA. At this stage, the synapses are not stable, and most SV packets move dynamically along the axons. Therefore, we measured endocytic kinetics at sites where the SypHy signal increased robustly upon stimulation (300 APs at 10 Hz). In the control boutons, all exocytosed SVs were recovered by endocytosis. In the presence of BFA, however, SV recycling was incomplete, and only ~63% of the exocytosed SVs were retrieved (Fig. 3A,B). The inhibitory effect of BFA on endocytosis was more severe in more mature neurons. At DIV14 and DIV19, only ~56 and 39% of exocytosed SVs, respectively, were retrieved by endocytosis in the presence of BFA (Fig. 3C–F). Figure 3G shows that the ratio of the BFA insensitive fraction of endocytosis to the BFA sensitive fraction (the BIS/BS ratio) increases as neurons mature. This suggests that CIE is the major endocytic mode in mature neurons.

Plotting the SypHy decrease on a semi-log plot shows the differences in endocytic kinetics between the control and BFA-treated neurons at each stage of neuronal maturation. Because the SypHy signal did not decrease to the initial level in the BFA-treated neurons, we chose, for simplicity, to measure the rate constants for the decay to reach its final value. Again, for DIV9 neurons, endocytosis was well described by a single exponential decay (*k* = 0.023 s^−1^). A similar plot for BFA-treated neurons also yielded a single decay component, but this time with a rate constant of 0.011 s^−1^ (Fig. 3H). For DIV14 and 19 neurons, endocytosis in the control and BFA-treated neurons was also described by single exponential decays with a lower rate constant for the BFA-treated neurons than for the control neurons (control: *k* = 0.028 s^−^1 for DIV14; *k* = 0.037 s^−1^ for DIV19; BFA-treated: 0.008 s^−1^ for DIV14 and 0.006 s^−1^ for DIV19, Fig. 3I,J).

As an independent way to block CIE, we used roscovitine, a selective antagonist of CDK5, because inhibition of CDK5 is known to selectively reduce CIE during strong stimulation and to arrest endosome formation in nerve terminals without affecting the endocytosis of single SVs^22^. We found that pretreatment with roscovitine for 30 min indeed inhibited endocytic retrieval of SVs even after the first stimulation. As in the case of BFA treatment, the inhibitory effect of roscovitine was more severe in more mature neurons. At DIV9, ~61% of exocytosed SVs were endocytosed whereas only ~57% and ~44% were endocytosed at DIV14 and at DIV19, respectively (Supplemental Fig. 4A–C). The ratio of the roscovitine insensitive fraction of endocytosis to the roscovitine sensitive fraction (the RIS/RS ratio) increased as neurons matured, again indicating that CIE is the major endocytic mode in mature neurons (Supplemental Fig. 4D).

### FM 1–43 uptake confirms that CIE becomes the major endocytic pathway as neurons mature

Recent studies suggest that different SV proteins recycle by different mechanisms^23^,^24^. To avoid a possible bias caused by SV protein-specific recycling, we used FM 1–43.

**Figure 4:**
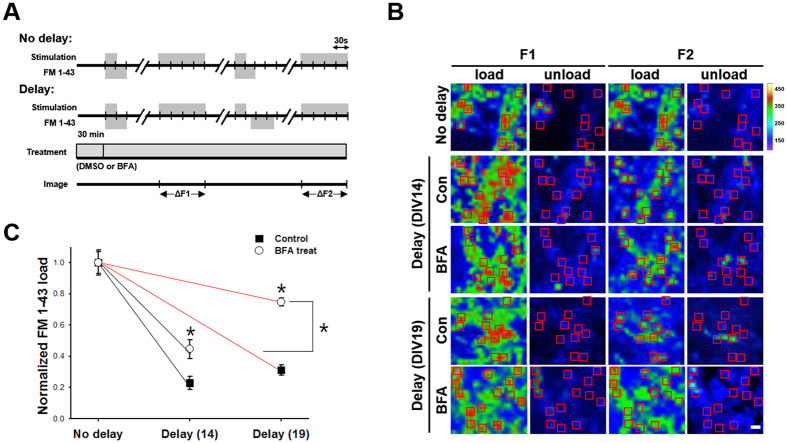
FM 1–43 uptake experiment verifies that BFA sensitive CIE becomes major endocytic pathway as neuronal maturation proceeds. **(A)** Neurons were treated with or without 10 μg/ml BFA and two consecutive loading/unloading cycles were performed with FM 1–43. First, neurons were loaded with FM 1–43 by field stimulation (300 APs at 10 Hz) and kept for an additional 30 s after the stimulation to label all endocytosed vesicles. After a 10-min resting period, a stimulus of 1,200 APs at 10 Hz was given to unload FM 1–43. After the first cycle, the second load was applied 50 s after the onset of stimulation to estimate the fraction of SVs that underwent endocytosis during 50 s. (**B)** Representative fluorescent images of two consecutive load-unload cycles with FM1-43. No delay or a 50 s-delay was given between the onset of stimulation and FM loading during the second cycle at DIV14 and DIV19, respectively. Rectangular regions of interest were drawn around the synaptic boutons, then average intensities were calculated. Right: heat-map for pseudo-colored fluorescence intensity. Scale bar: 1 μm. (**C)** FM 1–43 intensities of neurons at DIV14 (black) or DIV19 (red) are expressed as the ratio of the intensities in the first load (ΔF1) to those in the second load (ΔF2). The closed rectangle represents the changes of intensity ratio for boutons of the control neurons and the open circle represents the change of intensity ratio for boutons in the BFA-treated neurons (DIV14; Control: 1.0 ± 0.07 for no delay, 0.23 ± 0.04 for delay; BFA treatment: 1.0 ± 0.08 for no delay, 0.45 ± 0.06 for delay, DIV19; Control: 1.0 ± 0.05 for no delay, 0.31 ± 0.03 for delay; BFA treatment: 1.0 ± 0.03 for no delay, 0.75 ± 0.03 for delay). DIV14: *n* = 201 neurons from 12 independent coverslips for control, *n* = 162 neurons from 9 independent coverslips for BFA treatment. DIV19: *n* = 109 neurons from 6 independent coverslips for control, *n* = 73 neurons from 4 independent coverslips for BFA treatment. Data are presented as means ± SEM *p < 0.01 (Student’s *t*-test).

After a first loading and unloading cycle of FM 1–43 with 300 APs at 10 Hz, a second loading was applied 50 s after the onset of electrical stimulation. During that 50 s interval, some vesicles underwent endocytosis and escaped from being labeled, *i.e.,* the lower the endocytic capacity, the higher the intensity of FM 1–43 staining^25^. We found that the intensity of FM 1–43 staining of the BFA-treated synaptic boutons was invariably higher than that of the control boutons at DIV14, and it was increased further at DIV19 (Fig. 4A–C). These results indicate that the contribution of the BFA insensitive fraction of endocytosis decreases during strong stimulation as neuron mature, confirming that CIE is the major endocytic pathway in mature neurons.

### CIE retrieves SVs through an AP-1/AP-3-dependent mechanism

AP-1 and AP-3 are known to be essential for SV retrieval by CIE^7^,^8^. In addition, AP-1/σ1B-deficient mice showed decreased SV reformation and endosome accumulation^9^. Defects in SV recycling and biogenesis were also observed in mice lacking both AP-3A and AP-3B^7^,^11^. To assess the roles of AP-1 or AP-3 on SV retrieval mode, we generated small hairpin RNAs (shRNAs) targeting AP-1 or AP-3. Suppression of AP-1 or AP-3 expression by shRNAs was confirmed by immunofluorescence staining of AP-1 or AP-3 in shRNA-transfected neurons, in which AP-1 or AP-3 immunoreactivity is markedly abolished to ~20% (AP-1) and ~10% (AP-3), of the control levels, respectively (Supplemental Fig. 5).

**Figure 5:**
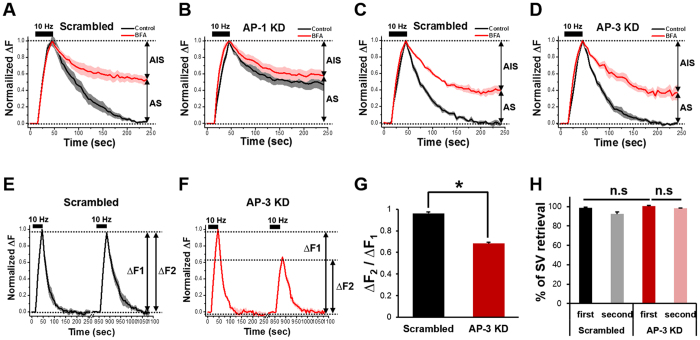
BFA-sensitive SVs are retrieved via AP-1/AP-3 dependent mechanisms. (**A–D)** Hippocampal neurons were transfected with shRNAs targeted to AP-1 or AP-3 or respective scrambled shRNAs. At DIV14, neurons were stimulated with 300 APs at 10 Hz in the presence or absence of BFA, and average SypHy fluorescence intensity profile of the boutons was normalized to its peak value and plotted against time. Error bars indicate SEM. AP-1 sensitive SV (AS) = 49.0 ± 6.5%, AP-1 insensitive SV (AIS) = 51.0 ± 6.5% for AP-1 KD (AP-1 shRNA: *n* = 124 neurons from 14 independent coverslips for the scrambled, *n* = 89 neurons from 9 independent coverslips for AP-1 KD; AP-3 shRNA: *n* = 60 neurons from 7 independent coverslips for the scrambled, *n* = 73 neurons from 10 independent coverslips for AP-3 KD). (**E,F)** Average SypHy fluorescence intensity profiles of the boutons of the scrambled (black) or AP-3 knock-down (KD) neurons (red). The same group of neurons was stimulated with 300APs at 10 Hz twice and the change in fluorescence induced by the second stimulation (ΔF2) was normalized to that induced by the first stimulation (ΔF1). Error bars indicate SEM (*n* = 41 neurons from 5 independent coverslips for the scrambled, *n* = 28 neurons from 4 independent coverslips for AP-3 KD). (**G)** The ratio of ΔF2 over ΔF1. *p < 0.01 (Student’s *t*-test). (**H**) The percentage of SV retrieval over fusion after the first and second stimulation in the scrambled (black, grey) and AP-3 KD neurons (red, pink). Data are presented as means ± SEM.

We found that neurons transfected with small hairpin RNA (shRNA) for AP-1 had marked defects in SV retrieval comparable to those observed with BFA (Fig. 5A), and there was no additional effect of BFA on their retrieval (Fig. 5B). These results indicate that the knockdown of AP-1, like BFA, not only affects the reformation of SVs from endosomes but also blocks the budding of bulk endosomes from the plasma membrane.

Interestingly, when we transfected neurons with the shRNA of AP-3, the exocytosed SVs were fully retrieved after stimulation (Fig. 5C,D). A double stimulation experiment revealed that in the case of AP-3 knockdown, endocytosis after the first stimulation was not affected while the peak amplitude of SypHy fluorescence during the second stimulation was approximately halved compared to the first stimulation (Fig. 5E–H). These results suggest that AP-3 only functions in SV reformation from the endosome, while AP-1 also affects bulk endosome formation from the plasma membrane.

### The extent of CIE during SV endocytosis is regulated by neuronal activity

Because the contribution of CIE to endocytic retrieval grows with neuronal maturation, we hypothesized that an important factor contributing to this effect was the history of chronic synaptic activity during maturation. We tested this hypothesis by measuring recycling in DIV14 neurons whose neuronal activity was decreased chronically by exposure to tetrodotoxin (TTX) for 48 h. We rationalized that neurons at DIV14 are “newly matured neurons” and highly active and thus can be affected more profoundly by activity modulation.

**Figure 6:**
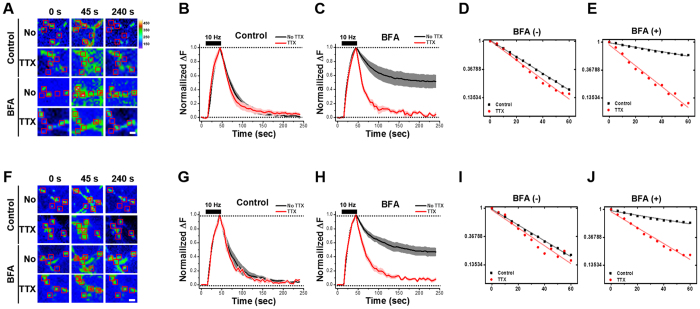
The extent of BFA-sensitive SV retrieval is regulated by either chronic or acute changes in neuronal activity. Neurons at DIV 14 were treated with TTX for 48 h (**A–E**) or 30 min (**F-J**). Representative fluorescent time-lapse images of presynaptic boutons of SypHy transfected neurons after either chronic (**A**) or acute (**F**) TTX pretreatment followed by a stimulus of 300 APs at 10 Hz in the presence or absence of 10 μg/ml BFA (acute: *n* = 202 neurons from 16 independent coverslips for No TTX, *n* = 41 neurons from 5 independent coverslips for TTX; chronic: *n* = 287 neurons from 15 independent coverslips for No TTX, *n* = 128 neurons from 14 independent coverslips for TTX). Rectangular regions of interest were drawn around the synaptic boutons, then average intensities were calculated. Right: heat-map for pseudo-colored fluorescence intensity. Scale bar: 1 μm. Average SypHy fluorescence intensity profiles of the respective boutons (**B,C** and **G,H**). Semilog plot of each of the traces in the TTX-treated neurons with treatment time of 48 h **(D,E;** *k* = 0.029 s^−1^ for control; *k* = 0.034 s^−1^ for control with TTX) and 30 min **(I,J;** *k* = 0.011 s^−1^ for BFA only; *k* = 0.026 s^−1^ for BFA with TTX). TTX pretreatment completely abolished BFA-induced inhibitory effect on endocytosis. Data are presented as means ± SEM.

Chronic pretreatment with TTX itself did not affect the SV retrieval (Fig. 6A,B,D) but did completely abolish the inhibitory effect of BFA on endocytosis (Fig. 6A,C,E), indicating that the history of chronic synaptic activity during neuronal maturation influences the extent of CIE.

We wondered whether an acute, rather than a chronic, change of synaptic activity would also modulate the extent of CIE. To test this possibility, we pretreated SypHy-transfected neurons at DIV14 with TTX for 30 min. This acute pretreatment with TTX did not affect SV retrieval (Fig. 6F,G,I) whereas BFA slowed the rate of endocytosis (Fig. 6F,H,J). As in the case of chronic treatment, acute treatment completely abolished the BFA-sensitivity of endocytosis (Fig. 6F,H,J), showing that the acute as well as chronic history of synaptic activity during neuronal maturation affects the extent of CIE.

## Discussion

The question of whether SVs are all retrieved directly from the plasma membrane has been debated for a long time. Although numerous studies suggest that the majority of SV retrieval occurs through CME^3^,^26^,^27^, in which AP-2 is thought to act as a hub molecule^3^,^28^,^29^, recent studies combining various genetic and optical tools have provided concrete evidence for the existence of a process for bulk SV biogenesis from endosomal intermediates.

The molecular mechanism of CIE is not fully understood although some key components have recently been identified. Intense stimulation triggers a substantial calcium influx which activates calcineurin, the calcium-calmodulin dependent protein phosphatase^30^. This dephosphorylates several dephosphins^4^,^31^,^32^. Dephosphorylated dynamin is essential for CIE^33^,^34^. It is induced by intense stimulation and interacts with syndapin and triggers CIE^33^. Interestingly, while other key SV endocytic proteins such as amphiphysin, endophilin, and SNX9 interact with dynamin 1 regardless of its phosphorylation state, syndapin interacts specifically with the dephosphorylated form of dynamin 1^25^,^35^,^36^.

In the present work, we used BFA to prevent SV reformation from endosomes via CIE. Most previous studies, however, have suggested that BFA does not arrest the formation of endosomes during CIE but does stop the subsequent budding of SVs from them. Moreover, we anticipated that arresting budding would not prevent re-acidification of the endosomes. Why then did we see an incomplete recovery of the SypHy response (i.e., incomplete loss of SypHy fluorescence)? We considered two explanations: either BFA also inhibits the reacidification of the endosomes or it inhibits their budding off from the plasma membrane. As reported above, we found that the latter explanation is supported through three independent experimental approaches using acidification, TEV protease and QSY35 quenching.

We also found that the fraction of BFA-sensitive endosome-mediated CIE during strong stimulation increased with neuronal maturation and rose to 2/3 of the SV endocytosis in fully mature neurons. Because CIE is triggered when neuronal activity increases and CME is saturated^2^,^5^,^17^, this increasing contribution of CIE seems understandable in view of the fact that as neurons mature, they need a fast, high-capacity SV retrieval mechanism to deal with the intense network activity. We found, however, that the extent of CIE is not fixed because either chronic or acute neuronal inactivity induced by TTX led to a shift in favor of CME (Fig. 6). Thus, our results indicate that mature neurons use the CME and CIE pathways to different extents depending on the synaptic activity; this may result in activity-dependent changes in the SV recycling mechanism that influence transmitter release and contribute to synaptic plasticity.

The sensitivity of nerve terminals to BFA is likely due to an effect of BFA on AP-1/AP-3^7^,^10^. A recent study using AP-1/σ1B-deficient mice showed that AP-1/σ1B have a role in the disassembly of bulk endosomal structures suggesting that they act during SV recycling through endosomes^9^. In addition, AP-2 knockdown led only to a slowing, not a cessation, of endocytosis with the remaining component relying mostly on AP-1^29^. Thus, AP-1 may have a role in the bulk CIE mechanism. Our results also show that AP-1 inhibition selectively inhibited CIE because it eliminated the BFA effect (Fig. 5A,B). AP-3 was originally implicated in SV biogenesis in PC-12 cells which are sensitive to BFA^10^. A recent study showed that the interaction of vesicular glutamate transporter 1 (VGLUT1) with endophilin accounts for its recycling through a pathway involving AP-2; however, after prolonged stimulation, the AP-3 pathway retrieves VGLUT1 indicating that these two pathways are being used to different extents depending on the activity level^8^. Interestingly, we found that although AP-1 and AP-3 both have a role during CIE, AP-3 only has a role in SV reformation from the endosome while AP-1 additionally affects bulk endosome formation from the plasma membrane. In summary, our findings strongly indicate that SV recycling depends heavily on an endosomal route that requires the AP-1/AP-3 complex.

Ultrafast endocytosis retrieves excess membrane via synaptic endosome ~100 ms after a single brief stimulation at physiological temperature, and SVs are regenerated through those endosomes ~6 s after the stimulation. Interestingly, at room temperature, clathrin regenerates SV from the surface rather than from the synaptic endosome^14^. At first glance, ultrafast endocytosis seems highly relevant to CIE; however, a direct comparison is deceptive because the current study was done with intense stimulation rather than just a single or brief stimulation and reported on the activity-dependent switch between CME and CIE rather than on the temperature-dependent switch. Whether ultrafast endocytosis also functions during intense stimulation and whether temperature is also responsible for shifting from CME to CIE are certainly interesting topics that require further study.

Another recent study has shown that CME is not essential for SV retrieval at the plasma membrane during high frequency stimulation. Instead, CIE involving endosome-like vacuole invagination via dynamin 1/2 and endophilin occurs at the presynaptic membrane whereas clathrin and AP-2 mediated SV formation occurs from internal endosomal structures^12^. This study is highly relevant to our study because both report on the activity-dependent changes in the SV recycling mechanism between CME and CIE. Our data, however, show that beside CIE, CME also occurs during intense stimulation although its contribution becomes smaller as neurons mature. Nevertheless, these results strongly support the conclusion that most SVs may be regenerated from endosomes during intense stimulation although how precisely neuronal activity regulates the choice between CME and CIE requires further study.

## Methods

### Ethics statement

Animal experimental procedures were approved by the Institute of Animal Care and Use Committee (IACUC, Approval ID number: SNU-100930-5) of Seoul National University, Korea. All experiments were carried out in accordance with the approved guidelines and regulations.

### Materials

Brefeldin A (BFA) was from Epicentre (Madison, WI) and bafilomycin A1 was from Calbiochem (San Diego, CA). AcTEV was from Invitrogen (Invitrogen, San Diego, CA, USA) and QSY35 was Thermo Fisher Scientific (Waltham, MA). Dimethyl sulfoxide (DMSO), tetrodotoxin (TTX), and other chemicals were from Sigma (St. Louis, MO).

### Neuron culture and transfection

Hippocampal neurons derived from E-18 pregnant Sprague Dawley female rats were prepared as described^37^. Briefly, hippocampi were dissected, dissociated with papain and triturated with a polished half-bore Pasteur pipette. The cells (2.5 × 10^5^) in minimum Eagle’s medium (MEM) supplemented with 0.6% glucose, 1 mM pyruvate, 2 mM L-glutamine, 10% fetal bovine serum and antibiotics were plated on poly-D-lysine-coated glass coverslips in 60-mm Petri dishes. Four hours after plating, the medium was replaced with Neurobasal media (Invitrogen) supplemented with 2% B-27 and 0.5 mM L-glutamine. 4 μM of 1-β-D-cytosine-arabinofuranoside (Ara-C, Sigma) was added as needed. Neurons were transfected using a modified calcium-phosphate method^38^. Briefly, 6 μg of cDNA and 9.3 μl of 2 M CaCl_2_ were mixed in distilled water to a total volume of 75 μl, and the same volume of 2x borate buffered saline was added. The cell culture medium was completely replaced by transfection medium (MEM, 1 mM pyruvate, 0.6% glucose, 10 mM glutamine, and 10 mM N-2-hydroxyl piperazine-N′-2-ethane sulfonic acid (HEPES), pH 7.65). Subsequently, the cDNA mixture was added and the cells were incubated in a 5% CO_2_ incubator for 90 min. The cells were washed twice with washing medium (pH 7.4) and then returned to the original culture medium.

### Immunocytochemistry

Cultured neurons were fixed in 4% paraformaldehyde, 4% sucrose, PBS for 15 min at RT and subsequently permeabilized with 0.25% Triton X-100 in PBS for 3 min at RT or in 100% Methanol at −20 °C for 5 min. Neurons were then blocked with 10% normal goat serum in PBS for 1 hour at RT. Primary antibodies diluted in 3% normal goat serum in PBS were added and incubated for 2 h at RT. Secondary antibodies (Invitrogen) were diluted 1:2000 in 3% normal goat serum in PBS and incubated for 1 h at RT. Mouse monoclonal anti-γ adaptin antibody was purchased from BD Biosciences. Anti-AP-3δ antibody was from Developmental Studies Hybridoma Bank (Iowa City, IA). Secondary antibodies were from Jackson ImmunoResearch (West Grove, PA).

### Synaptophysin-pHluorin endocytosis assay

Coverslips transfected with synaptophysin-pHluorin (SypHy) were mounted in a perfusion/stimulation chamber equipped with platinum-iridium field stimulus electrodes (EC-S-10, LCI, Seoul, Korea) on the stage of an Olympus IX-71 inverted microscope with 60 X, 1.35 NA oil lens (Olympus, Tokyo, Japan). The cells were continuously perfused at room temperature with Tyrode solution. 10 μM 6-cyano-7-nitroquinoxaline-2,3-dione (CNQX) and 50 μM of DL-2-amino-5-phosphonovaleric acid (AP-V) were added to the Tyrode solution to reduce spontaneous activity and to prevent recurrent excitation during stimulation. Time-lapse images were acquired every 5 s for 4 min using a back-illuminated Andor iXon 897 EMCCD camera (Andor Technologies, Belfast, Northern Ireland) driven by MetaMorph Imaging software (Molecular Devices, Sunnyvale, CA). Neurons were stimulated by passing current through two parallel platinum wires which lie just above the plane of the cover slip using an A310 Accupulser current stimulator (World Precision Instruments, Sarasota, FL). The settings on the stimulator connected to the platinum wires were established beforehand using Fluo-4 loaded neurons. By applying 0.2 Hz stimulation during time-lapse imaging, we monitored calcium signals (Fluo-4) and gradually increased currents until the optimum stimulus strength were found. A general criterion is that each electrical pulse should generate action potentials reliably without damaging the cell. Our typical settings were 1 ms bipolar pulses of 50–80 mA in a volume of 250 μl Tyrode solution. Quantitative measurements of the fluorescence intensity at individual boutons were obtained by averaging pixel intensities of a selected area using Image J (NIH). Individual regions were selected by hand and rectangular regions of interest were drawn around the synaptic boutons, then average intensities were calculated. Large puncta, which are typically interpreted as clusters of smaller synapses, were excluded from the selection procedure. The center of intensity of each synapse was calculated to correct for any image shifts over the course of the experiment. Fluorescence was expressed in intensity units that correspond to fluorescence values averaged over all pixels within the region of interest. Net fluorescence changes were obtained by subtracting the average intensity of the first four frames (*F*_0_) from the intensity of each frame (*F*_*t*_) for individual boutons. They were then normalized to the maximum fluorescence intensity (*F*_max_−*F*_0_) and averaged. All fitting was done using individual error bars to weight the fit, using Origin 8.6 (OriginLab Corporation, Northampton, MA). For the double stimulation experiment, neurons transfected with SypHy were stimulated with 300 APs at 10 Hz, then re-stimulated with the same condition after 5 min (10 min for AP-3 KD experiment). Time-lapse images were acquired every 5 s for 10 min. The detailed information regarding experimental setup and procedures can be found elsewhere^39^,^40^ (See also Supplemental Fig. 2).

### Drug treatment

Time-lapse images were acquired from the SypHy-transfected neurons that were stimulated at 10 Hz, 300 APs. Afterwards, the cells were incubated with 10 μg/ml BFA or 100 μM roscovitine for 30 min at 37 °C. During imaging, BFA or roscovitine was included in Tyrode solution (136 mM NaCl, 2.5 mM KCl, 2 mM CaCl_2_, 1.3 mM MgCl_2_, 10 mM HEPES, 10 mM glucose, pH 7.4). To test the effect of neuronal activity, neurons were treated with 1 μM TTX for 48 h at DIV14 until DIV16 (chronic effect) or for only 30 min at DIV14 (acute effect).

### Tobacco etch virus (TEV) protease and QSY35 treatment

AcTEV protease recognizes the 7 amino-acid sequence (Glu-Asn-Leu-Tyr-Phe-Gln-Gly) and cleaves between Gln and Gly. The synaptobrevin-2-TEV-pHluorin (synaptobrevin-TEV-pHluorin) expression construct, in which a DNA fragment encoding the cleavage site for recombinant TEV protease, was introduced into the linker region between the synaptobrevin-2 and the pHluorin moiety that was kindly provided by Dr. Robert Edwards at UCSF. 5 min after stimulation, synaptobrevin-TEV-pHluorin transfected neurons were treated with 60 unit AcTEV protease and 2 mM dithiothreitol and imaged every 5 s for 8.5 min. For QSY35 treatment, SypHy transfected neurons were treated with 10 μM QSY35 after stimulation and imaged every 5 s for 8.5 min.

### FM 1–43 endocytosis assay

Two consecutive load–unload cycles were performed with FM 1–43^25^. FM 1–43 was loaded with the onset of stimulation (300 APs at 10 Hz) and held for 1 min further after stimulation to label all endocytosed vesicles. After 10 min resting period, 1,200 APs at 10 Hz stimulus was given to unload FM 1–43. Net fluorescence changes were obtained by subtracting the intensity gained in the unloaded image from the intensity in the loaded image. The second load was added 50 s after the onset of stimulation for 1 min to estimate the fraction of SVs that had undergone endocytosis during that period.

### Reacidification assay

The re-acidification kinetics of the endocytosed SVs were measured using SypHy transfected neurons. The extracellular solution was changed from pH 7.4 to pH 5.5 and back to pH 7.4 to measure the surface expression level of SypHy. After stimulation with 300 APs at 10 Hz, the extracellular solution was changed from pH 7.4 to pH 5.5 for quenching all surface SypHy then back to pH 7.4. In order to measure the total SV pool, neurons were treated with NH_4_Cl solution.

### Protein knockdown by shRNA

The shRNA for AP-1 γ subunit (rat), AP-3 δ subunit (rat) were designed from following oligonucleotides. shRNA AP-1, 5′-GCGCCTGTACAAGGCAATT-3′; scrambled AP-1, 5′- GCGTACGGACCTAGTTACA-3′; shRNA AP-3, 5′-ACAAAGTGTTCCTCAAGTA-3′; scrambled AP-3, 5′-GATGTCTACAACACTAGTA-3′. Complementary oligonucleotides were synthesized separately, with the addition of an ApaI site at the 5′ end and an EcoRI site at the 3′ end. The annealed cDNA fragment was cloned into the ApaI-EcoRI sites of pSilencer.U.1.0 vector (Ambion) modified by inserting DsRed sequence at the C terminus. Neurons at DIV 7 ~ 9 were transfected with SypHy, treated with shRNA or scrambled vectors at DIV 14 and incubated for 72 h before the assay.

### Statistical analysis

All data are expressed as mean  ±  SEM using Origin 8.6 software. To assess statistical significance between two groups, the Kolmogorov-Smirnov test was performed, and p values were determined by the unpaired Student’s *t*-test. For multiple conditions, one-way ANOVA followed by Tukey’s HSD post analysis was used. “*n*” stands for the number of independent coverslips. Detailed parameters for each experiments were given in Supplemental Information.

## Additional Information

**How to cite this article**: Park, J. *et al.* Endosome-mediated endocytic mechanism replenishes the majority of synaptic vesicles at mature CNS synapses in an activity-dependent manner. *Sci. Rep.* **6**, 31807; doi: 10.1038/srep31807 (2016).

## Supplementary Material

Supplementary Information
