# Supplementary material for: Endosome-mediated endocytic mechanism replenishes the majority of synaptic vesicles at mature CNS synapses in an activity-dependent manner
Source: Sci Rep. 2016 Aug 18;6:31807. doi: 10.1038/srep31807 (PMC4989163; doi:10.1038/srep31807)
Supplement: Supplementary Information [file srep31807-s1.pdf]

Endosome-mediated endocytic mechanism replenishes the majority of synaptic vesicles at mature CNS synapses in an activity-dependent manner

Joohyun Park, Oh Yeon Cho, Jung Ah Kim and Sunghoe Chang

SUPPLEMENTAL FIGURE LEGENDS

Supplemental Figure 1. A, Hippocampal neurons at DIV19 were undergone two consecutive FM 1-43 dye loading/unloading cycles. Neurons were loaded with FM 1–43 by field stimulation (300 APs at 10 Hz) and kept for an additional 30 s after the stimulation to label all endocytosed vesicles. After a 10 min-resting period, a 1,200 APs at 10 Hz stimulus was given to unload FM 1–43. After the first round of the cycles, neurons were treated with DMSO for 30 min. **B,** Representative traces showing the average unloading responses of the control group in each first and second cycle. The extent of SV turnover was estimated from the total amount of unloaded dye in each first (ΔF1) and second cycle (ΔF2). FM1-43 signals were normalized to the intensity recorded in the first cycle (*n* = 29 neurons from 3 independent coverslips). Error bars indicate SEM. **C**, The amount of unloaded dye from each first (ΔF1) and second cycle (ΔF2) over the first cycle. Data are presented as means ± SEM, n.s. = not significant.

Supplemental Figure 2. A, Schematic figure showing synaptic vesicle recycling. At rest, SypHy fluorescence is quenched by the intraluminal acidic pH of the vesicle (1). Upon stimulation, vesicles fuse with the plasma membrane and expose their lumen to the neutral pH of the extracellular medium (pH ~ 7.4), causing an increase in SypHy fluorescence (2). The

fluorescence is then quenched once again after endocytosis (3) and reacidification (4, 5). **B**, Representative fluorescent time-lapse images of SypHy transfected neurons at DIV19 that were stimulated with 300 APs at 10 Hz. Individual regions were selected by hand and rectangular regions of interest were drawn around the synaptic boutons, then average intensities were calculated. Large puncta, which are typically interpreted as clusters of smaller synapses, were excluded from the selection procedure. Net fluorescence changes were obtained by subtracting the average intensity of the first four frames (F_0) from the intensity of each frame (F_t) for individual boutons. Representative images were selected for display from the entire time series; pre-stimulation, peak of response and post-stimulation. Right: Heat-map for pseudo-colored fluorescence intensity. Each of the numbers at the bottom corresponds to each stage in **A.** Scale bar: 5 μm. **C,** Changes in fluorescence intensity in response to electrical stimulation (300 APs at 10Hz, thick black bar) at individual synapses enclosed by rectangles from the image in **B**. Average of all displayed synapses is shown in red. **D,** Fluorescence intensity profile of each bouton was normalized to its peak value and plotted against time. Average of all displayed synapses is shown in red. Numbers at the bottom correspond to each stages in **A**.

Supplemental Figure 3. A, C, Schematic figures for two scenarios. Scenario-1, BFA does not block the budding of bulk endosomes from the plasma membrane but inhibits their subsequent re-acidification. Scenario-2, besides its effect on SV budding from the endosome, BFA actually blocks the budding of bulk endosomes from the plasma membrane (that is, the inside of the bulk endosomes should be accessible from outside the cell). **B,** Schematic figure for re-acidification experiments If scenario-1 is true, then ΔF1 (before stimulation) and ΔF2 (after stimulation) should be the same in the BFA-treated neurons (ΔF1 = ΔF2). If scenario-2 is true,

a second application of pH 5.5 after stimulation should decrease SypHy fluorescence all the way to zero ($\Delta F1 < \Delta F2$). **D,** Schematic figures for TEV cleavage and QSY35 quenching experiments. If scenario-1 is true, TEV protease or QSY35 should not have access to the cytosol of the bulk endosomes; thus, the resulting amount of fluorescence loss in the BFA-treated neurons should be the same as that in the control neurons ($\Delta F_{Con} = \Delta F_{BFA}$). If scenario-2 is true, TEV protease or QSY35 could cleave or quench the ecliptic pHluorin not only in the plasma membrane but also inside the bulk endosomes, and ΔF_{BFA} should be larger than ΔF_{Con}.

Supplemental Figure 4. The roscovitine-sensitive SV retrieval becomes the major pathway with neuronal maturation. A-C, Average SypHy fluorescence intensity profiles of the boutons from DIV9 (**A**), DIV14 (**B**) and DIV19 (**C**) neurons before (black) or after (red) 30 min treatment of 100 μM roscovitine (Ros), a CDK5 inhibitor, followed by a 300 APs at 10 Hz stimulation. Note that regardless of maturation, the SVs retrieval in Ros-treated neurons is impaired, although the degrees of sensitivity differ in a similar manner with BFA-treated neurons (*n* = 69 neurons from 4 independent coverslips for DIV9, n= 186 neurons from 4 independent coverslips for DIV14, *n* = 95 neurons from 4 independent coverslips for DIV19). **D,** The ratio of Ros-insensitive SV (RIS) / Ros-sensitive SV (RS): 1.74 ± 0.18 for DIV9, 1.48 ± 0.07 for DIV14, and 0.67 ± 0.04 for DIV19 neurons. Data are presented as means ± s.e. * $p < 0.05$ (ANOVA and Tukey's HSD post hoc test)

Supplemental Figure 5. shRNA-targeting AP-1 or AP-3 efficiently depletes AP-1 or AP-3 in hippocampal neurons, respectively. A, Primary hippocampal neurons transfected with or without shRNA-tageting AP-1 or AP-3 were stained with anti-AP-1 or AP-3 antibody followed

by Alexa 488-conjugated secondary antibody. Arrowheads indicate the cell bodies of shRNA-transfected cells. Expression levels of AP-1 and AP-3 were measured at cell bodies to avoid possible spatial overlap with other cells. Scale bar: 20 μm. **B,** In cells transfected with shRNAs, AP-1 or AP-3 was severely depleted compared with nontransfected cells: Expression levels over the nontransfected; AP-1 KD: 0.20 ± 0.06 (*n* = 25) whereas AP-3 KD: 0.13 ± 0.06 (*n* = 20). ***p<0.001(Student's *t*-test).

Supplemental Fig. 1

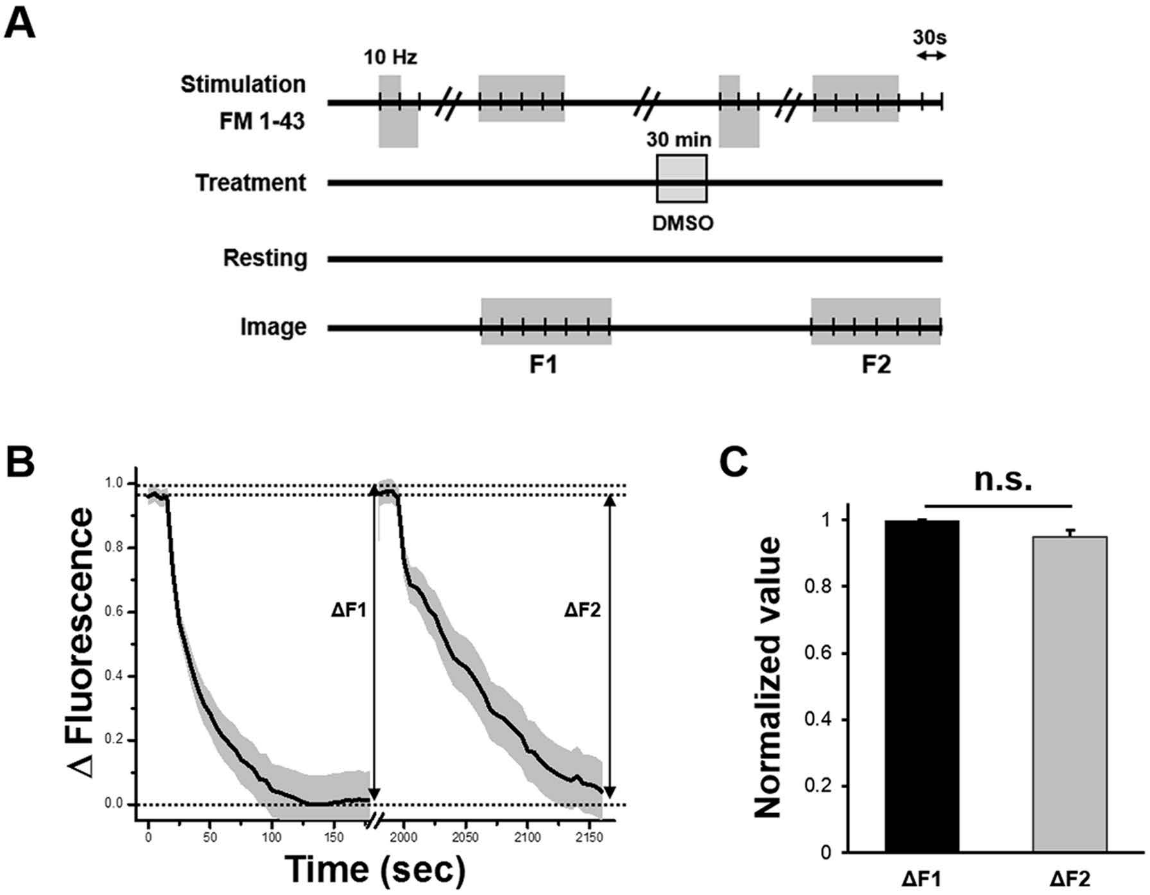

Supplemental Fig. 2

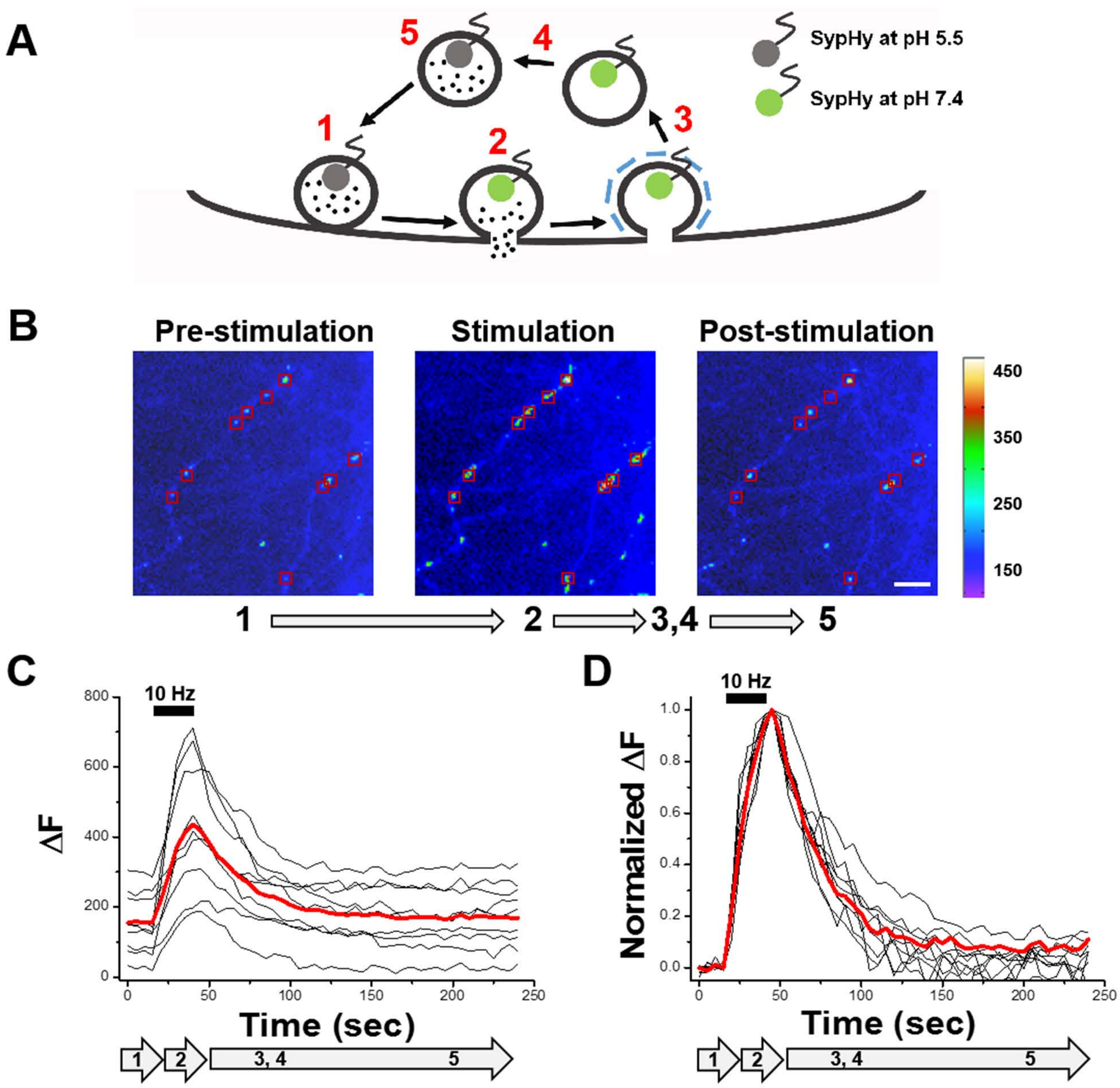

Supplemental Fig. 3

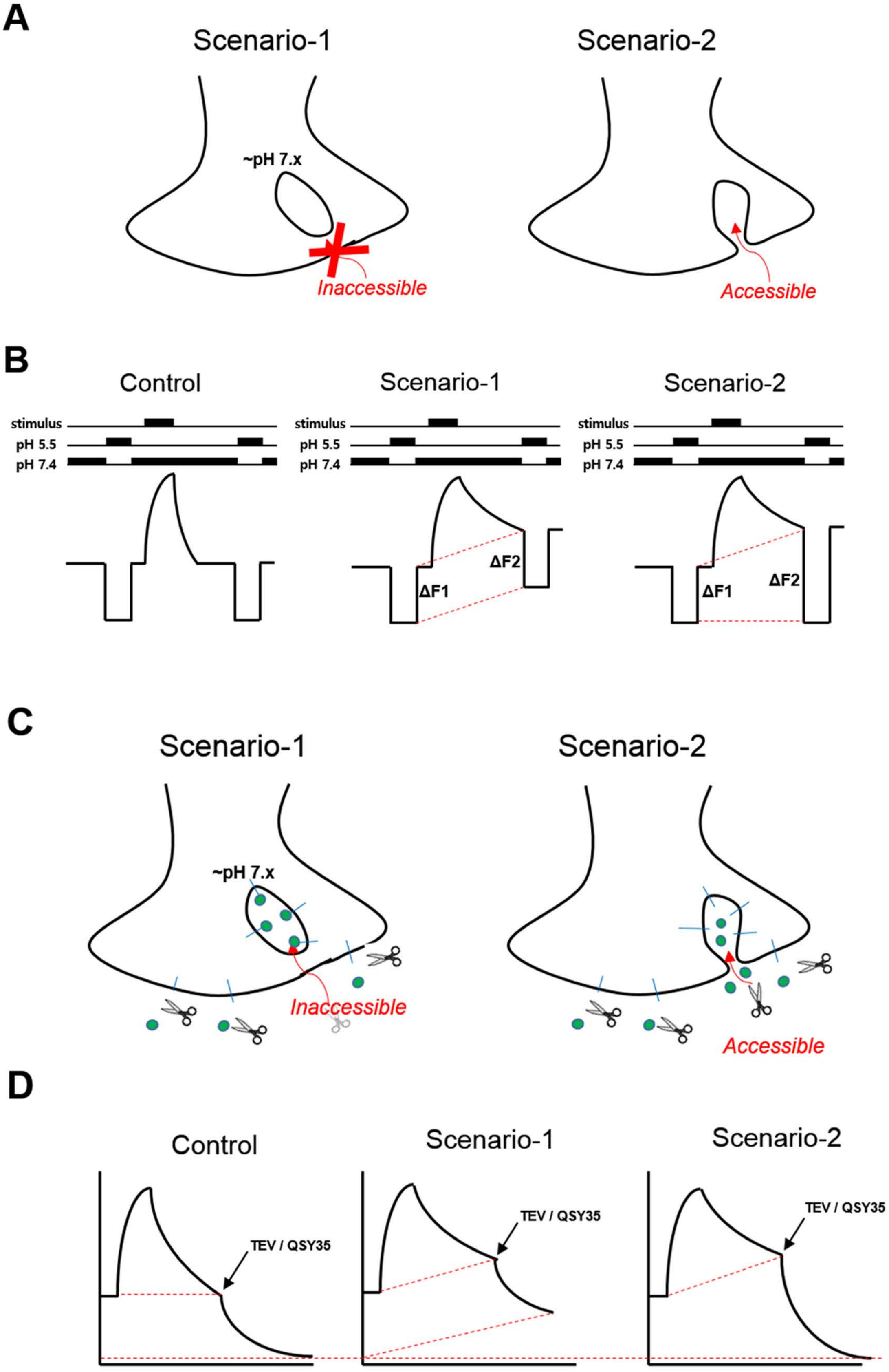

Supplemental Fig. 4

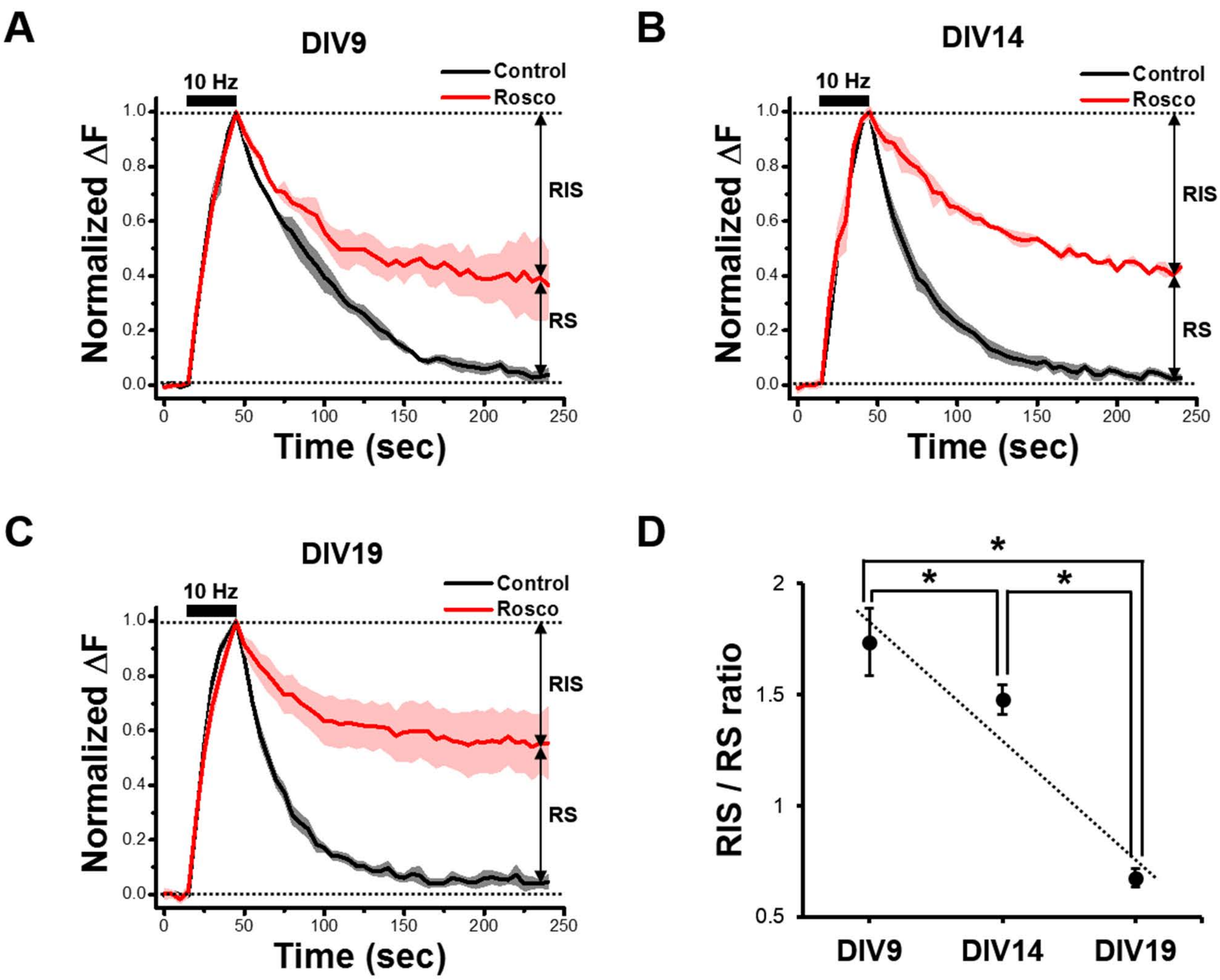

Supplemental Fig. 5

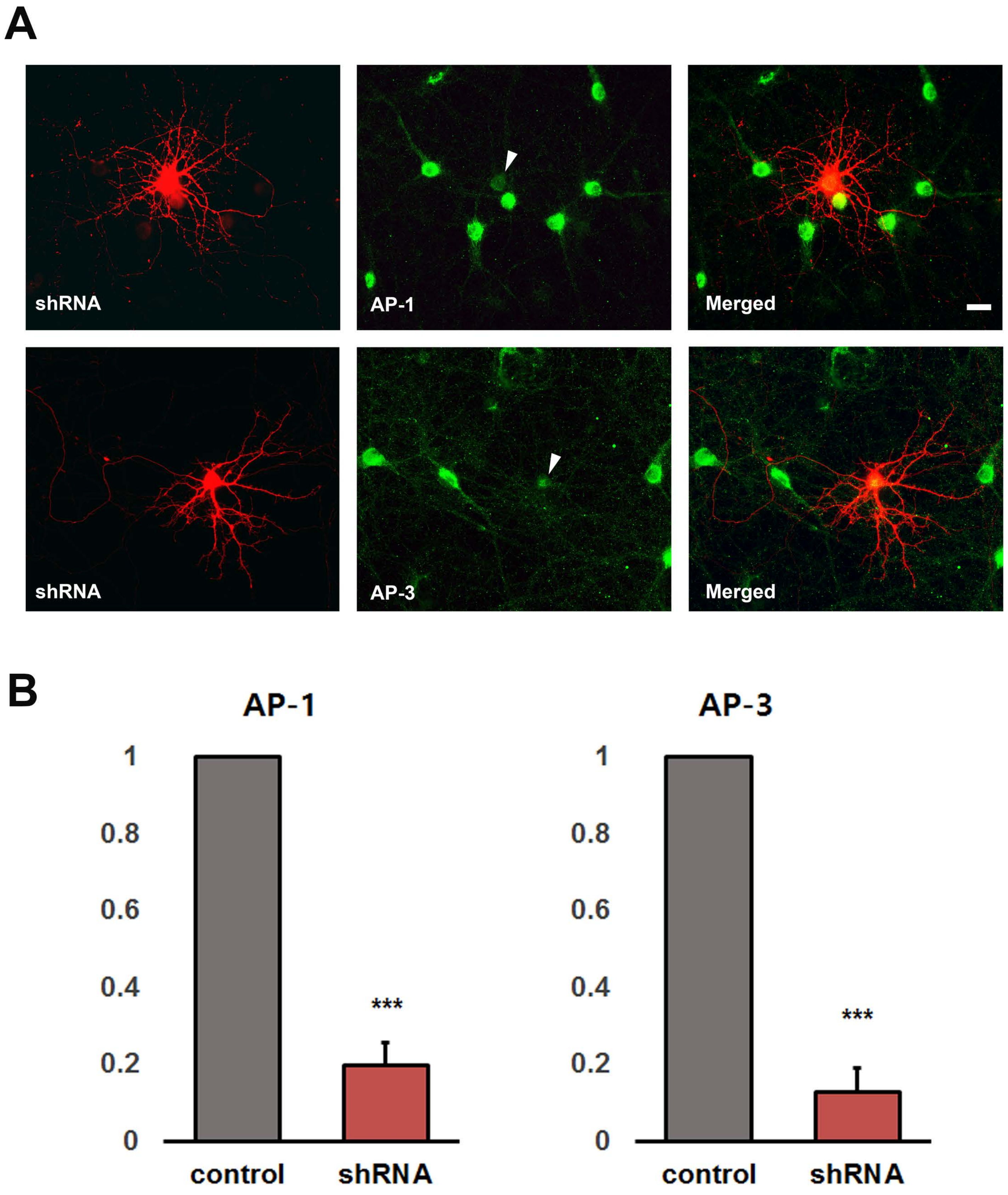

Statistical Parameters used in the Figures

Fig. 1D

Kolmogorov_Smirnov

normality test (mean)

	DF	Statistic	p-value	Decision at level(5%)
B	9	0.1721	1	Can't reject normality
C	9	0.19444	0.8996	Can't reject normality
D	9	0.15428	1	Can't reject normality

Control	dF1
BFA-1	dF2
BFA-2	dF3

mean +/- SEM

	N Analysis	N Missing	Mean	Standard Deviation	SE of Mean
B	9	0	1.07357	0.09762	0.03254
C	9	0	0.59879	0.19339	0.06446
D	9	0	0.59435	0.28571	0.09524

One-way ANOVA

	DF	Sum of Squares	Mean Square	F Value	Prob>F
Model	2	1.36527	0.68263	15.92967	3.96E-05
Error	24	1.02847	0.04285		
Total	26	2.39374			

	R-Square	Coeff Var	Root MSE	Data Mean
	0.57035	0.27398	0.20701	0.75557

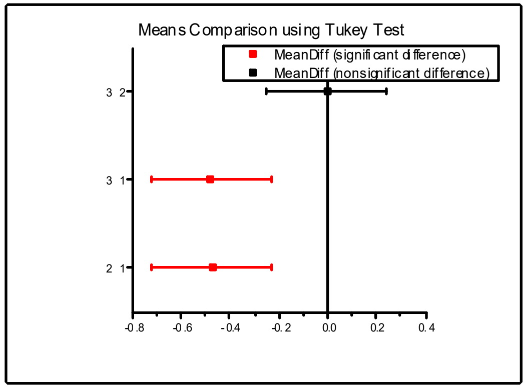

P value

	MeanDiff	SEM	q Value	Prob	Alpha	Sig	LCL	UCL
2 1	-0.47478	0.09759	6.88051	1.67E-04	0.05	1	-0.71847	-0.23108
3 1	-0.47922	0.09759	6.94496	1.49E-04	0.05	1	-0.72292	-0.23553
3 2	-0.00445	0.09759	0.06445	0.99886	0.05	0	-0.24814	0.23925

Fig. 1F

Kolmogorov_Smirnov

normality test (mean)

	DF	Statistic	p-value	Decision at level(5%)
B	9	0.14472	1	Can't reject normality
C	6	0.19453	1	Can't reject normality

**Control**
**BFA**

mean +/- SEM

	N Analysis	N Missing	Mean	Standard Deviation	SE of Mean
B	9	0	0.94332	0.046	0.01533
C	6	2	0.60572	0.2448	0.09994

P value 0.001240931

Fig. 1G

Kolmogorov_Smirnov

normality test (mean)

	DF	Statistic	p-value	Decision at level(5%)
1	9	0.16422	1	Can't reject normality
2	9	0.22468	0.7012	Can't reject normality
3	6	0.33926	0.40464	Can't reject normality
4	6	0.25105	0.81277	Can't reject normality

Control-first
Control-second
BFA-first
BFA-second

mean +/- SEM

	N Analysis	N Missing	Mean	Standard Deviation	SE of Mean
1	9	0	0.01825	0.02588	0.00863
2	9	0	0.06449	0.07672	0.02557
3	6	0	0.45315	0.13409	0.05474
4	6	0	0.14318	0.11526	0.04705

One-way ANOVA

	DF	Sum of Squares	Mean Square	F Value	Prob>F
Model	3	0.77265	0.25755	32.07436	6.85E-09
Error	26	0.20877	0.00803		
Total	29	0.98142			

	R-Square	Coeff Var	Root MSE	Data Mean
	0.78727	0.62189	0.08961	0.14409

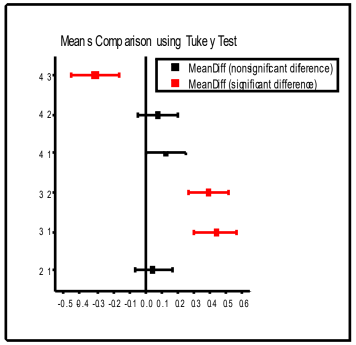

P value

	MeanDiff	SEM	q Value	Prob	Alpha	Sig	LCL	UCL
2 1	0.04624	0.04224	1.54806	0.69586	0.05	0	-0.06964	0.16212
3 1	0.4349	0.04723	13.0227	8.50E-08	0.05	1	0.30534	0.56446
3 2	0.38866	0.04723	11.63808	6.83E-08	0.05	1	0.2591	0.51822
4 1	0.12492	0.04723	3.74079	0.06172	0.05	0	-0.00463	0.25448
4 2	0.07868	0.04723	2.35616	0.36123	0.05	0	-0.05087	0.20824
4 3	-0.30997	0.05174	8.47319	1.43E-05	0.05	1	-0.4519	-0.16805

Fig. 2C

Kolmogorov_Smirnov

normality test (mean)

	DF	Statistic	p-value	Decision at level(5%)
B	5	0.20579	1	Can't reject normality
C	5	0.34104	0.5086	Can't reject normality

**Control**
**BFA**

mean +/- SEM

	N Analysis	N Missing	Mean	Standard Deviation	SE of Mean
B	5	0	0.92089	0.28221	0.12621
C	5	0	2.66035	1.51307	0.67667

P value 0.03541777

Fig. 2E

Kolmogorov_Smirnov

normality test (mean)

	DF	Statistic	p-value	Decision at level(5%)
B	4	0.16139	1	Can't reject normality
C	3	0.20569	1	Can't reject normality

**Control**
**BFA**

mean +/- SEM

	N Analysis	N Missing	Mean	Standard Deviation	SE of Mean
B	4	1	0.39637	0.12038	0.06019
C	3	2	0.60439	0.07865	0.04541

P value 0.049608725

Fig. 2G

Kolmogorov_Smirnov

normality test (mean)

	DF	Statistic	p-value	Decision at level(5%)
B	4	0.35462	0.59918	Can't reject normality
C	3	0.26939	1	Can't reject normality

**Control**
**BFA**

mean +/- SEM

	N Analysis	N Missing	Mean	Standard Deviation	SE of Mean
B	4	0	0.1728	0.12927	0.06464
C	3	0	0.68033	0.13313	0.07686

P value 0.00383386

Fig. 3G

Kolmogorov_Smirnov

normality test (mean)

	DF	Statistic	p-value	Decision at level(5%)
B	4	0.16052	1	Can't reject normality
C	4	0.26002	0.98095	Can't reject normality
D	4	0.19681	1	Can't reject normality

DIV9
DIV14
DIV19

mean +/- SEM

	N Analysis	N Missing	Mean	Standard Deviation	SE of Mean
B	4	0	1.88025	0.06515	0.03258
C	4	0	1.35516	0.01483	0.00742
D	4	0	0.82964	0.01167	0.00584

One-way ANOVA

	DF	Sum of Squares	Mean Square	F Value	Prob>F
Model	2	2.20754	1.10377	719.62913	1.18E-10
Error	9	0.0138	0.00153		
Total	11	2.22134			

	R-Square	Coeff Var	Root MSE	Data Mean
	0.99379	0.0289	0.03916	1.35502

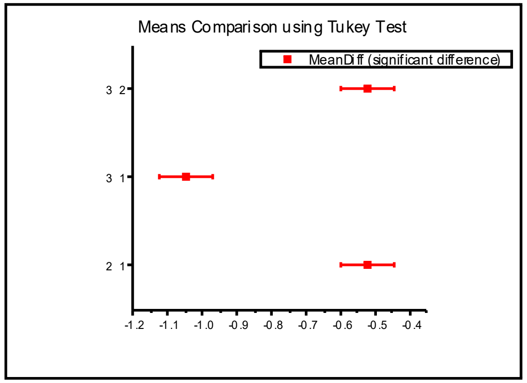

P value

	MeanDiff	SEM	q Value	Prob	Alpha	Sig	LCL	UCL
2 1	-0.52509	0.02769	26.81502	2.18E-08	0.05	1	-0.60241	-0.44777
3 1	-1.0506	0.02769	53.65181	0.00E+00	0.05	1	-1.12792	-0.97329
3 2	-0.52552	0.02769	26.83678	2.15E-08	0.05	1	-0.60283	-0.4482

Fig. 4C

Kolmogorov_Smirnov

normality test (mean)

	DF	Statistic	p-value	Decision at level(5%)
B	12	0.18132	0.81272	Can't reject normality
C	9	0.22187	0.71817	Can't reject normality
D	6	0.22709	0.94462	Can't reject normality
E	4	0.24222	1	Can't reject normality

DIV14-control-delay
DIV14-BFA-delay
DIV19-control-delay
DIV19-BFA-delay

mean +/- SEM

	N Analysis	N Missing	Mean	Standard Deviation	SE of Mean
1	12	0	0.23284	0.04316	0.01246
2	9	0	0.44871	0.14237	0.04746
3	6	0	0.32103	0.18186	0.07424
4	4	0	0.76264	0.44003	0.22001

One-way ANOVA

	DF	Sum of Squares	Mean Square	F Value	Prob>F
Model	3	0.90886	0.30295	8.80611	3.10E-04
Error	27	0.92887	0.0344		
Total	30	1.83773			

	R-Square	Coeff Var	Root MSE	Data Mean
	0.49456	0.48689	0.18548	0.38094

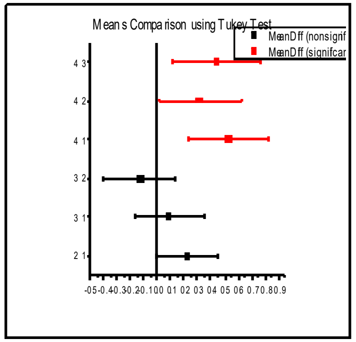

P value

	MeanDiff	SEM	q Value	Prob	Alpha	Sig	LCL	UCL
2 1	0.21587	0.08179	3.73267	0.06167	0.05	0	-0.00795	0.43969
3 1	0.08819	0.09274	1.34483	7.78E-01	0.05	0	-0.1656	0.34198
3 2	-0.12768	0.09776	1.84715	5.67E-01	0.05	0	-0.3952	0.13983
4 1	0.5298	0.10709	6.99669	1.95E-04	0.05	1	0.23675	0.82285
4 2	0.31393	0.11146	3.98317	0.04192	0.05	1	0.00891	0.61894
4 3	0.44161	0.11973	5.21633	5.22E-03	0.05	1	0.11397	0.76925

Fig. 5G

Kolmogorov_Smirnov

normality test (mean)

	DF	Statistic	p-value	Decision at level(5%)
B	5	0.19308	1	Can't reject normality
C	4	0.21285	1	Can't reject normality

Control
AP-3 KD

mean +/- SEM

	N Analysis	N Missing	Mean	Standard Deviation	SE of Mean
B	5	0	0.96242	0.02752	0.01231
C	4	0	0.68398	0.02363	0.01182

P value 9.013E-07

Fig. 5H

Kolmogorov_Smirnov

normality test (mean)

	DF	Statistic	p-value	Decision at level(5%)
B	5	0.17633	1	Can't reject normality
C	5	0.32328	0.58233	Can't reject normality
D	4	0.23972	1	Can't reject normality
E	4	0.22173	1	Can't reject normality

Control-first
Control-second
AP-3KD-first
AP-3KD-second

mean +/- SEM

	N Analysis	N Missing	Mean	Standard Deviation	SE of Mean
1	5	0	98.67478	1.89719	0.84845
2	5	0	97.45195	2.19715	0.9826
3	4	0	100.46346	1.1276	0.5638
4	4	0	98.20448	0.92997	0.46499

One-way ANOVA

	DF	Sum of Squares	Mean Square	F Value	Prob>F
Model	3	21.11961	7.03987	2.45682	1.06E-01
Error	14	40.1162	2.86544		
Total	17	61.23581			

	R-Square	Coeff Var	Root MSE	Data Mean
	0.34489	0.01716	1.69276	98.62808

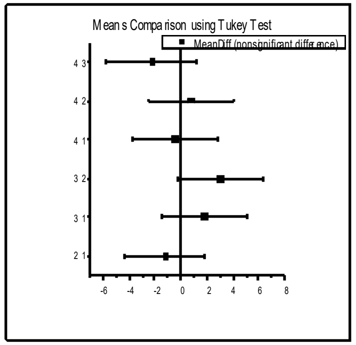

P value

	MeanDiff	SEM	q Value	Prob	Alpha	Sig	LCL	UCL
2 1	-1.22283	1.0706	1.61531	0.67076	0.05	0	-4.33458	1.88892
3 1	1.78868	1.13554	2.22765	4.23E-01	0.05	0	-1.51182	5.08919
3 2	3.01152	1.13554	3.75058	7.92E-02	0.05	0	-0.28899	6.31202
4 1	-0.47029	1.13554	0.58571	0.97514	0.05	0	-3.7708	2.83021
4 2	0.75254	1.13554	0.93722	0.90929	0.05	0	-2.54797	4.05305
4 3	-2.25898	1.19696	2.66899	2.77E-01	0.05	0	-5.73802	1.22006

Supplemental Fig. 1C

Kolmogorov_Smirnov

normality test (mean)

	DF	Statistic	p-value	Decision at level(5%)
B	3	1.23E-128	2.39E+263	Can't reject normality
C	3	0.29891	0.99138	Can't reject normality

Control- first
Control- second

mean +/- SEM

	N Analysis	N Missing	Mean	Standard Deviation	SE of Mean
B	3	0	1	0	0
C	3	0	0.9502	0.03937	0.02273

P value 0.093552554

Supplemental Fig. 4D

Kolmogorov_Smirnov

normality test (mean)

	DF	Statistic	p-value	Decision at level(5%)
B	4	0.16175	1	Can't reject normality
C	4	0.25502	1	Can't reject normality
D	4	0.25191	1	Can't reject normality

DIV9
DIV14
DIV19

mean +/- SEM

	N Analysis	N Missing	Mean	Standard Deviation	SE of Mean
B	4	0	1.73557	0.15144	0.07572
C	4	0	1.47753	0.06564	0.03282
D	4	0	0.87701	0.03435	0.01717

One-way ANOVA

	DF	Sum of Squares	Mean Square	F Value	Prob>F
Model	2	1.55244	0.77622	81.93118	1.68E-06
Error	9	0.08527	0.00947		
Total	11	1.63771			

	R-Square	Coeff Var	Root MSE	Data Mean
	0.94794	0.07139	0.09733	1.36337

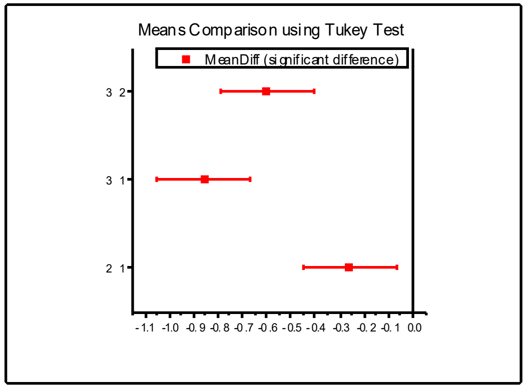

P value

	MeanDiff	SEM	q Value	Prob	Alpha	Sig	LCL	UCL
2 1	-0.25805	0.06883	5.30222	1.14E-02	0.05	1	-0.45021	-0.06588
3 1	-0.85856	0.06883	17.64139	1.44E-06	0.05	1	-1.05072	-0.6664
3 2	-0.60051	0.06883	12.33917	2.93E-05	0.05	1	-0.79268	-0.40835
